# Comparison of Clinical Outcomes of Definitive and Postoperative Radiotherapy for Adenoid Cystic Carcinoma of the Head and Neck: Can Definitive Radiotherapy Be a Treatment Option?

**DOI:** 10.3390/cancers13215507

**Published:** 2021-11-02

**Authors:** Nobutaka Mizoguchi, Kio Kano, Satoshi Shima, Keisuke Tsuchida, Yosuke Takakusagi, Itsuko Serizawa, Keiko Akahane, Masahiro Kawahara, Manatsu Yoshida, Yuka Kitani, Kaori Hashimoto, Madoka Furukawa, Tadashi Kamada, Hiroyuki Katoh, Daisaku Yoshida, Katsuyuki Shirai

**Affiliations:** 1 Department of Radiation Oncology, Kanagawa Cancer Center, Yokohama 241-8515, Japan; mizoguchin@kcch.jp (N.M.); k-kanou@kcch.jp (K.K.); s-shima@kcch.jp (S.S.); ketsuchi@kcch.jp (K.T.); y-takakusagi@kcch.jp (Y.T.); itsuko.serizawa@kcch.jp (I.S.); kamada.tadashi@qst.go.jp (T.K.); hkatoh@kcch.jp (H.K.); d.yoshida@kcch.jp (D.Y.); 2 Department of Radiology, Jichi Medical University Saitama Medical Center, Saitama 330-8503, Japan; keiko-a@omiya.jichi.ac.jp (K.A.); masahr.kawa@gmail.com (M.K.); 3 Department of Head and Neck Surgery, Kanagawa Cancer Center, Yokohama 241-8515, Japan; m-yoshida@kcch.jp (M.Y.); y-kitani@kcch.jp (Y.K.); k-hashimoto@kcch.jp (K.H.); furukawam@kcch.jp (M.F.); 4 Department of Radiation Oncology, Jichi Medical University Hospital, Tochigi 329-0498, Japan

**Keywords:** adenoid cystic carcinoma, head and neck cancer, definitive radiotherapy, postoperative radiotherapy, surgical resection

## Abstract

**Simple Summary:**

Adenoid cystic carcinoma of the head and neck is a rare malignant tumor; thus, it is difficult to establish an optimal treatment based on clinical trials with a large number of enrolled patients. Retrospective analyses of a small number of cases have revealed that the standard treatment is surgical resection followed by postoperative radiotherapy, while definitive radiotherapy is considered inadequate. Previous studies have used classical techniques for radiotherapy and did not evaluate the efficacy of current radiotherapy techniques, which may have underestimated the efficacy of definitive radiotherapy. We retrospectively analyzed 44 cases of adenoid cystic carcinoma of the head and neck treated with current radiotherapy techniques. Our results show that definitive radiotherapy is comparable to surgical resection followed by postoperative radiotherapy with respect to overall survival and local control. The results suggest that definitive radiotherapy can be an effective treatment option for adenoid cystic carcinoma of the head and neck.

**Abstract:**

Background: The standard treatment for adenoid cystic carcinoma of the head and neck is surgical resection followed by postoperative radiotherapy (PORT). Currently, definitive radiotherapy (defRT) is considered an inadequate treatment; however, its data are based on studies using classical radiotherapy techniques. Therefore, the therapeutic effects of current radiotherapy techniques have not been adequately evaluated, and it may have underestimated the efficacy of defRT. Methods: We retrospectively analyzed 44 adenoid cystic carcinoma patients treated with radiotherapy based on modern treatment techniques from 1993 to 2017. Results: Twenty-four patients underwent PORT and 20 patients underwent defRT. The 5-year overall survival rates for patients treated with PORT and defRT were 85.3% and 79.7%, respectively. The 5-year local control rates were 82.5% and 83.1%, respectively. There were no statistically significant differences in the overall survival and local control of patients treated with PORT and defRT (*p* = 0.4392 and *p* = 0.0904, respectively). Conclusion: Our results show that defRT is comparable to surgical resection followed by PORT with respect to overall survival and local control. The results suggest that defRT can be an effective treatment option for adenoid cystic carcinoma of the head and neck.

## 1. Introduction

Adenoid cystic carcinoma is reported to have an annual incidence of 3 to 4.5 per million and accounts for ~1% of all head and neck malignancies and ~10% of all major salivary gland tumors [1,2,3]. According to the Surveillance, Epidemiology, and End Results database, the incidence of adenoid cystic carcinoma in the United States has declined over the past several decades [4]. An indolent but aggressive clinical course, an infiltrative local growth pattern, a propensity for perineural invasion, and frequent local recurrence characterize adenoid cystic carcinoma. In addition, it is prone to hematogenous metastasis, mainly in the lungs, although lymph node metastasis is not frequent [5,6,7]. Since adenoid cystic carcinoma is a rare disease, only a few studies have focused on clinical and pathological prognostic factors. According to previous studies, risk factors for head and neck adenoid cystic carcinoma include positive resection margins, perineural invasion, vascular invasion, and solid type pathology [8,9,10,11,12]. Although there have been no large-scale clinical trials, surgical resection followed by postoperative radiotherapy (PORT) is considered as the standard treatment, whereas definitive radiotherapy (defRT) is considered insufficiently effective [13,14,15,16,17]. The analysis that resulted in defRT not being considered the standard treatment involved many cases based on classical irradiation treatment techniques, which likely underestimated the efficacy of defRT. With the development of radiotherapy techniques, it is now possible to deliver high doses to tumors while reducing the dose to normal tissues. It is necessary to evaluate defRT using modern treatment techniques. The purpose of this study is a retrospective analysis of the treatment effects and adverse events associated with PORT and defRT using modern radiotherapy techniques for adenoid cystic carcinoma of the head and neck.

## 2. Materials and Methods

### 2.1. Patients and Methods

The analysis included 44 patients who were histologically diagnosed with adenoid cystic carcinoma of the head and neck and underwent radiotherapy at our hospital from August 1993 to August 2017. The UICC TNM classification (8th edition) was used for staging. The pathological classification of adenoid cystic carcinoma is divided into solid histological and non-solid histological subtypes [18]. In this study, we also classified the subtypes into those with and without solid components. Our treatment strategy was to resect the tumor if it was considered medically completely resectable and if the patient was judged to be functionally and cosmetically acceptable for resection. PORT was recommended for essentially all patients. Meanwhile, in cases that were medically unresectable or refusal of surgery, we administered defRT. All of the surgical cases were performed at our institution. The surgical type and extent of resection were determined according to the primary tumor, with complete removal of gross and microscopic lesions whenever possible.

### 2.2. Radiotherapy

#### 2.2.1. Postoperative Radiotherapy

The tumor bed was defined as the clinical target volume (CTV). In cases with perineural invasion, the area along the neural tracts to the skull base and peripheral site was also included as CTV. Planning target volume (PTV) was set at a margin appropriate for the CTV to account for setup error. The prescribed dose was 60 Gy in 30 fractions to PTV for patients with negative resection margins and 66 Gy in 33 fractions to PTV for patients with positive resection margins.

#### 2.2.2. Definitive Radiotherapy

Gross tumor volume (GTV) was determined based on computed tomography (CT), magnetic resonance imaging (MRI) images, and clinical findings. A margin of 10–20 mm was added to the GTV to define the CTV. In cases of suspected neural invasion, an additional margin was added to the area along the neural tract to the skull base. Prophylactic irradiation of the regional lymph nodes was not performed. PTV was defined as the CTV with an appropriate margin set to account for setup error. The prescribed doses ranged from 66 Gy in 33 fractions to 70 Gy in 35 fractions.

### 2.3. Evaluation

Physical examinations were performed once a month for 1 year after the end of treatment followed by once every 3 months for 2–3 years, once every 6 months for 4–5 years, and once a year thereafter. Imaging studies included CT or MRI every 3–6 months for the first 2 years after treatment and every 6–12 months thereafter. Treatment effects were assessed using the revised response evaluation criteria in solid tumors (RECIST) guideline (version 1.1). Acute and late adverse events were assessed using the Common Terminology Criteria for Adverse Events (CTCAE) version 5.0.

### 2.4. Statistical Analyses

The cumulative incidences of overall survival rate (OS), local control rate (LC), distant metastasis-free survival rate (DMFS), and progression-free survival rate (PFS) were evaluated using the Kaplan–Meier method. The starting date for follow-up was defined as the start date of radiotherapy. Histological subtype, primary site, tumor stage, and nodal stage were evaluated as potential risk factors for OS and LC. All statistically significant (*p* < 0.05) factors on univariate analysis were evaluated using the Cox proportional hazards model. P-values less than 0.05 were considered statistically significant, and all statistical tests were 2-sided. These statistical tests were performed with the assistance of GraphPad Prism version 9 software (GraphPad Software, Inc., San Diego, CA, USA).

## 3. Results

### 3.1. Characteristics of the Patients

**Figure 1:**
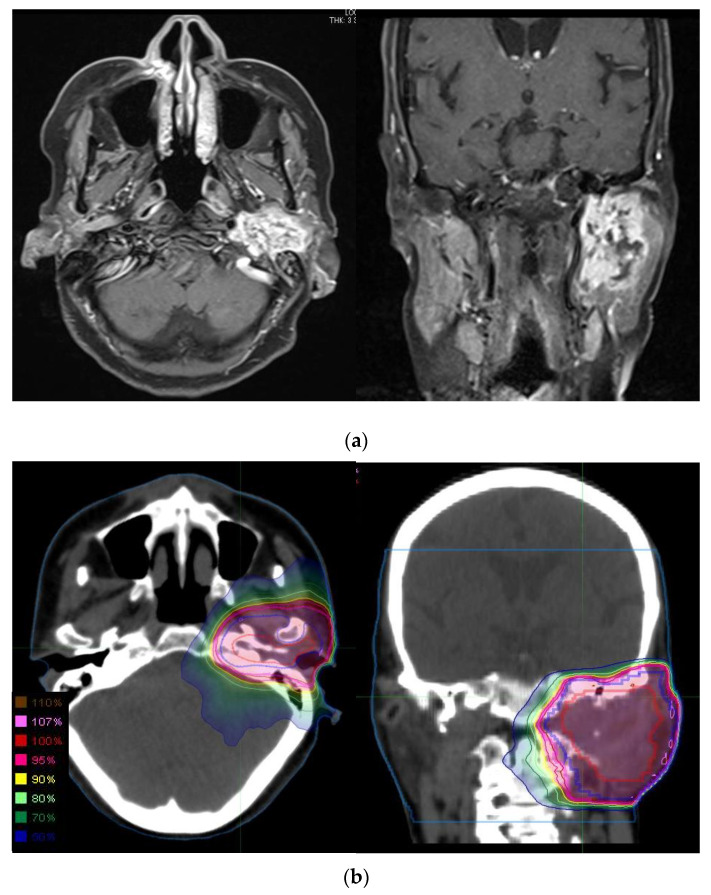
Representative case of parotid gland adenoid cystic carcinoma treated with defRT using IMRT. The 75-year-old woman with left parotid gland cancer, cT4bN0M0. Left facial nerve disorder was observed. (**a**) MRI contrast-enhanced T1-weighted images revealed the parotid gland tumor with the extension to base of skull. The patient refused surgery and hoped to receive defRT. (**b**) Dose distribution of defRT using 66 Gy in 33 fractions. The GTV, CTV, and PTV are shown in red, blue, and magenta, respectively. CTV margin was extended to base of the skull considering perineural invasion. PTV margin was added to CTV to account for patient motion and the field margins. Abbreviations: defRT, definitive radiotherapy; IMRT, intensity-modulated radiotherapy; MRI, magnetic resonance imaging; GTV, gross tumor volume; CTV, clinical target volume; and PTV, planning target volume.

A total of 44 patients with a diagnosis of primary adenoid cystic carcinoma of the head and neck received radiation therapy at the Kanagawa Cancer Center. Patient characteristics are summarized in Table 1, and representative case is shown in Figure 1. The median follow-up time was 76 months (range, 9–220 months) for all patients, 60 months (range, 9–220 months) for PORT cases, and 91 months (range, 13–210 months) for defRT cases. The median age of the PORT cases was 57 years (range, 38–73), and the median age of the defRT cases was 63 years (range, 16–76). Gender was slightly more female. Performance status was good in all patients. Histological types with solid components were observed in 10 PORT cases and two defRT cases. The primary sites included 16 salivary glands, 11 oral cavities, 10 nasal or paranasal sinuses, six pharynxes, and one lacrimal sac. Resection margins were microscopically positive in 16 patients and close-margin in 3 patients. No patients had gross residual disease after surgery. Neck dissection was not performed in any of the patients. PORT was performed in 24 patients and defRT in 20 patients. Major salivary gland carcinoma was significantly more common in patients treated with PORT (*p* = 0.0014). In PORT cases, T1, T2, T3, and T4 diseases were four, 13, five, and two patients, respectively, and in defRT cases, one, four, six, and nine, respectively. T4 cases were statistically significantly more common in the defRT cases (*p* = 0.0121). In PORT cases, N0, N1, and N2 diseases were 22, two, and 0 patients, respectively, and in defRT cases, 15, two, and three, respectively. In PORT cases, stage I, II, III, and IV diseases were four, 13, four, and three patients, respectively, and in defRT cases one, two, five, and 12, respectively. Stage IV cases were statistically significantly more common in the defRT cases (*p* = 0.0014). The median prescribed dose was 60 Gy (range, 60–80 Gy) for PORT cases and 66 Gy (range, 50–80 Gy) for defRT cases. CT simulation and treatment planning was performed for all cases. Of those, 13 patients underwent intensity-modulated radiotherapy (IMRT). Those included nine cases of PORT and four cases of defRT. Concurrent chemotherapy was administered in 22 cases, of which six were PORT cases and 16 were defRT cases. Concurrent chemotherapy was more common in defRT cases (*p* = 0.0007). In patients treated with PORT, the regimens included FP [5-fluorouracil/cisplatin (CDDP)] in four patients, CDDP in one patient, and CAP (cyclophosphamide, doxorubicin, and CDDP) in one patient. For the defRT cases, 14 patients were treated with FP and two patients with CDDP.

### 3.2. Overall Survival

**Figure 2:**
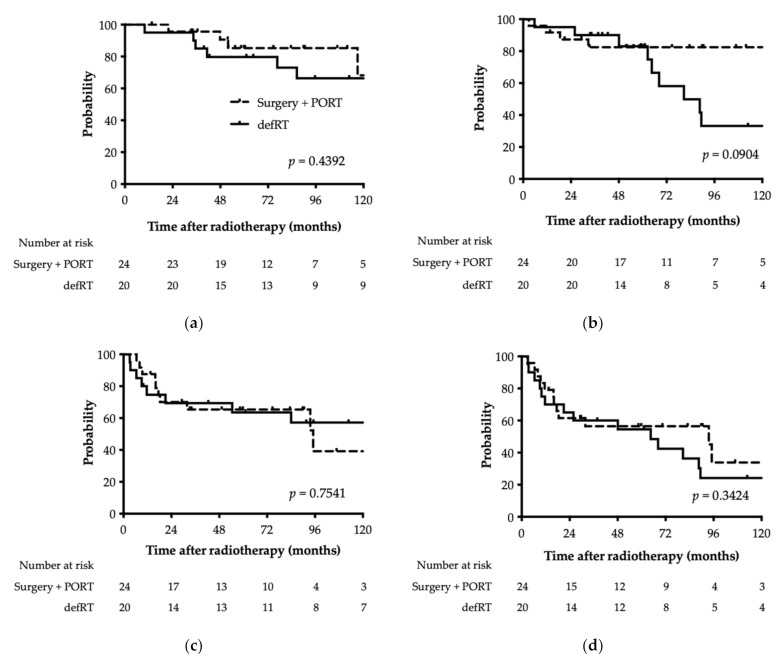
Kaplan–Meier plot of (**a**) overall survival, (**b**) local control, (**c**) distant metastasis, and (**d**) progression-free survival rate. The solid line indicates definitive radiotherapy, and the dashed line indicates surgical resection followed by postoperative radiotherapy. PORT, postoperative radiotherapy; defRT, definitive radiotherapy.

The OS for patients treated with PORT and defRT is shown in Figure 2a. Fourteen patients died; 13 died of primary disease, whereas the cause of death was unknown for one patient. Of the 13 deaths from primary disease, four were for PORT and nine for defRT. The OS at 3 and 5 years was 95.7% and 85.3% for PORT cases, respectively, and 85.0% and 79.7% for defRT cases. There was no statistically significant difference in OS between PORT and defRT cases (*p* = 0.4392).

### 3.3. Local Control

The LC for patients treated with PORT and defRT is shown in Figure 2b. Local recurrence was observed in 13 patients: four for PORT and nine for defRT. After 5 years, there were no local recurrences in PORT cases, whereas six patients developed local recurrence in defRT cases. The median time to local recurrence was 15 months for PORT cases (*n* = 4) and 65 months for defRT cases (*n* = 9). LC at 3 and 5 years was 82.5% and 82.5% for PORT cases, respectively, and 90.0% and 83.1% for defRT cases. There was no statistically significant difference in LC between PORT and defRT cases (*p* = 0.0904).

### 3.4. Distant Metastasis-Free Survival

The DMFS for patients treated with PORT and defRT is shown in Figure 2c. Distant metastasis was observed in 22 patients: 10 for PORT and 12 for defRT. The preferred site of distant metastasis was the lung (20 cases). DMFS at 3 and 5 years was 65.3% and 65.3% for PORT cases, respectively, and 69.3% and 63.6% for defRT cases. There was no statistically significant difference in DMFS between PORT and defRT cases (*p* = 0.7541).

### 3.5. Progression-Free Survival

The PFS for patients treated with PORT and defRT is shown in Figure 2d. Disease progression was observed in 27 patients: 12 for PORT and 15 for defRT. PFS at 3 and 5 years was 56.4% and 56.4% for PORT cases, respectively, and 60.0% and 54.5% for defRT cases. There was no statistically significant difference in PFS between PORT and defRT cases (*p* = 0.3424).

### 3.6. Risk Factors for Overall Survival and Local Control Rates

Risk factors for OS and LC were analyzed in all patients (Table 2). The statistically significant risk factors for OS were positive lymph nodes and stage IV cases (*p* < 0.0001 and *p* = 0.0022, respectively). Statistically significant risk factors for LC were T4 and stage IV cases (*p* = 0.0121 and *p* = 0.0062, respectively).

### 3.7. Comparison of Overall Survival and Local Control Rates by Treatment Modality in Patients with Clinical Factors

OS and LC by treatment method were compared in patients with each clinical factor (Table 3). There were no clinical factors that affect the OS. In T1/2/3 patients, PORT had statistically significantly better LC than defRT (*p* = 0.0397). In T4 and stage IV cases, defRT had statistically significantly better LC than PORT (*p* = 0.0004 and *p* = 0.0193, respectively).

### 3.8. Acute and Late Adverse Events

Acute and late adverse events are shown in Table 4. Acute adverse events included grade 3 mucositis in 5 patients (11.4%), and no grade 4 or higher adverse events. Late adverse events included grade 3 keratitis in 1 patient (2.3%) and no grade 4 or higher adverse events.

## 4. Discussion

We analyzed 44 cases of adenoid cystic carcinoma of the head and neck treated with radiotherapy at our institution. Twenty-four patients underwent PORT, and 20 patients underwent defRT. The 5-year OS and LC for the defRT cases were 79.7% and 83.1%, respectively, which were comparable to that of PORT. The results of our analysis suggest that defRT is an effective treatment option for adenoid cystic carcinoma of the head and neck.

Table 5 shows the results of treatment for adenoid cystic carcinoma of the head and neck by treatment modality. Previous studies have reported 5-year OS of 57–85% and LC of 56–86% for surgery alone for adenoid cystic carcinoma of the head and neck [14,15,16,17]. Postoperative radiotherapy improves OS and LC, which have been reported to be 75–80% and 73–94%, respectively [13,14,15,16,17]. In our analysis, the 5-year OS and LC for PORT cases were 85.3% and 82.5%, respectively, which were similar to that of previous reports.

Meanwhile, previous reports have shown that defRT is not associated with favorable outcomes, with 5-year OS ranging from 25% to 56% and 5-year LC ranging from 27% to 55% [13,14]. In this study, the 5-year OS and LC were 79.7% and 83.1%, respectively, which are favorable results for patients treated with defRT. There are at least two possible reasons for the inadequate results of defRT reported in previous studies. First, many patients were treated from the 1960s and classical radiotherapy techniques were used. Second, a large proportion of patients had advanced T3-T4 stage disease (73–86%, Table 5). Similar to previous reports, most of defRT cases of our study had advanced disease, such as T3-T4 (75%, Table 5). However, radiotherapy cases were performed between 1993 and 2017, and both PORT and defRT were performed using modern techniques.

In our study, there were significantly more T4 or stage IV diseases in defRT cases than in PORT cases (Table 1). Nevertheless, OS was not statistically different between defRT and PORT cases (*p* = 0.4392). There was also no statistically significant difference in LC (*p* = 0.0904). LC trended to decrease in defRT cases after 5 years. There was no local recurrence after 5 years in PORT cases, whereas local recurrence was observed after 5 years in 6 of 9 (67%) defRT cases. The median time to local recurrence in defRT cases was 65 months. For defRT cases, longer and more careful follow-up is considered necessary compared with PORT cases. In this analysis, LC was more favorable in T4 and stage IV diseases with defRT than PORT, which may result from the improvement of radiotherapy techniques.

Most reports suggest that the indication for postoperative irradiation is T3/4, positive lymph nodes, positive resection margins (R1/2 resection), and perineural invasion [8,10,11,12,15,16]. We compared the OS and LC of PORT and defRT cases with respect to each clinical factor (Table 3). PORT exhibited favorable LC for T1-3 cases, and defRT showed favorable LC for T4 cases (*p* = 0.0397 and *p* = 0.0004, respectively). Additionally, defRT exhibited better LC in stage IV diseases (*p* = 0.0193). The head and neck region is anatomically complex, and resection with adequate margins is difficult. It has been reported that 80% of cases involving the skull base had positive resection margins [19]. This may be one of the reasons for the inadequate therapeutic effect of surgery followed by postoperative radiotherapy in advanced T4 or stage IV cases. Meanwhile, in the case of defRT, it is necessary to reduce adverse events where the tumor is close to or invades important organs, such as the brain, brainstem, spinal cord, and mandible. In the era of classical radiotherapy, some cases may have experienced a reduction in the radiation dose to the tumor to avoid adverse events. Currently, IMRT, stereotactic radiotherapy, and particle therapy are available to intensively increase the radiation dose to the tumor while reducing adverse events for head and neck tumors [20,21,22]. With advances in treatment technology, definitive radiotherapy can contribute to improved OS and LC for adenoid cystic carcinoma of the head and neck. We used CT planning images as the basis for radiotherapy planning and included many cases of IMRT, which is now the standard treatment.

Among head and neck cancers, adenoid cystic carcinoma is more common in the major salivary glands [1,2]. In our study, 16 (36%) of 44 cases involved the major salivary glands. It accounted for 58% (14/24 patients) of PORT cases, which was significantly more than 10% (2/20 patients) of defRT cases (Table 1). Adenoid cystic carcinoma of the parotid gland has been reported to have a favorable prognosis compared with other head and neck regions since the mass is palpable and early detected, surgical access is easy, and complete resection with adequate margins is possible [1,23]. In our analysis, there was no apparent difference in OS and LC between PORT and defRT in major salivary gland cases (*p* = 0.7055 and *p* = 0.5490, respectively, Table 3).

Adenoid cystic carcinoma is classified as cribriform, tubular, solid, and mixed patterns based on histopathological characteristics, and many reports indicate that the prognosis is poor if the ratio of the solid pattern is high [18,24,25,26]. In our study, histological types with solid components tended to be more common in PORT cases (Table 1). However, the OS and LC of patients exhibiting a solid pattern were not statistically different between PORT and defRT (Table 3). Adenoid cystic carcinoma has a high rate of mixed subtypes, making it difficult to accurately classify by biopsy of only a portion of the tissue. A diagnosis of no solid pattern in defRT cases may include a solid component. Accurate pathological evaluation of defRT cases is difficult because of a lack of sufficient specimen volume.

Adenoid cystic carcinoma is considered a systemic therapy-resistant tumor. There have been several reports on chemotherapy for adenoid cystic carcinoma; however, its therapeutic efficacy is inadequate [27]. Although molecularly targeted therapies represent promising treatments, the response rate is low [28,29]. Since low somatic mutations and wide mutational diversity characterize adenoid cystic carcinoma [30,31], it is thought that there are few consistent factors that can be targeted by molecular targeted agents. Our analysis also revealed that concurrent chemotherapy did not improve OS or LC (Table 2). Concurrent chemotherapy tended to be more common in defRT cases (*p* = 0.007); however, there was no apparent difference in either OS or LC between PORT and defRT in cases involving chemotherapy (*p* = 0.8533 and *p* = 0.7894, respectively, Table 3). Although the response rate to chemotherapy and molecularly targeted therapy is low, the response rate to symptom improvement has been reported to be favorable [32]. The timing of drug therapy, especially in cases of recurrence and distant metastasis, should be carefully determined.

Treatment of adenoid cystic carcinoma with high-LET radiation has been reported for many years, and its favorable efficacy has been evaluated [33,34]. Recent report using fast neutron radiation showed favorable local control; however, rates of severe late adverse events were high [35]. Therefore, it is necessary to consider strategies to reduce adverse events. Carbon ions provide the best of both worlds, with an excellent dose distribution profile and the same radiobiological advantages as neutrons. Carbon-ion radiotherapy (CIRT) has been reported as a new therapeutic strategy for adenoid cystic carcinoma of the head and neck [36,37,38]. Carbon ions have a higher linear energy transfer resulting in a larger relative biological effectiveness than photons or protons. Moreover, the physical characteristics of carbon ions, owing to their ability to generate a spread-out Bragg peak, allow for an improved dose distribution. A multicenter retrospective study of 289 patients with adenoid cystic carcinoma of the head and neck in Japan reported 5-year LC and OS of 68% and 74%, respectively, indicating that CIRT is effective (Table 5). In Japan, CIRT for adenoid cystic carcinoma of the head and neck has been approved as an insured medical treatment. Because of the inadequate efficacy of definitive radiotherapy using photon beams for adenoid cystic carcinoma, particle radiotherapy has been actively used in unresectable or refused surgery cases. In our study, there was no significant difference in LC between PORT and defRT (*p* = 0.0904); however, the LC tended to gradually decrease in defRT cases after 5 years. Although long-term results with CIRT have not been reported, CIRT can be effective for long-term control.

CIRT is expected to have favorable LC; however, grade 3 or higher late adverse events (e.g., osteonecrosis of the jaw, visual impairment, and brain injury) have been reported in 15% (43/289 patients) of all patients [36]. In our analysis of 44 patients treated with photon radiotherapy, there were five (11%) grade 3 acute adverse events and no grade 4 or higher events. Of the late adverse events, there was one case (2%) of grade 3 and no cases of grade 4 or higher. Photon beam radiotherapy has a mild biological effect compared to CIRT, which could reduce the risk of late adverse effects. DefRT with photon beams is a treatment option for maintaining quality of life (QOL). In the case of defRT, it is necessary not only to provide LC but also to plan treatment to avoid QOL deterioration resulting from adverse events. It is essential to choose the treatment modality with careful consideration. Thus, if the results of defRT for adenoid cystic carcinoma of the head and neck are as favorable as those of surgical resection followed by PORT, it can be proposed as a treatment option to preserve function and appearance.

Our study has several limitations. First, it was a single-institution, retrospective analysis, and it had a small number of patients. Second, the characteristics of the patients treated with PORT and defRT were different. Therefore, the results of this study should be interpreted with caution.

Adenoid cystic carcinoma is a rare disease; thus, large-scale prospective clinical trials are difficult to conduct. Therefore, it would be appropriate to evaluate the role of defRT through the proposing of a standard protocol for selecting patients with specific characteristics at other centers in order to collect data from a more homogeneous cluster. A multicenter retrospective study, a meta-analysis using the results of previous studies, or a cross-sectional analysis using a multicenter database are warranted to identify which patients benefit from defRT.

**Table 5:** Comparison of treatment outcomes.

Authors	Year	Cases	Treatment Modality	Overall Survival (%)		Local Control (%)		Median Follow-Up Period (Year)	Ratio of T3/T4 (%)	Ratio of MSG Cancer (%)	Treatment Period
				5-Year	10-Year	5-Year	10-Year				
Iseli et al. [14]	2009	48	Surgery	85	51	72	42	9.0	40	45	1966–2007
Chen et al. [15]	2006	50		NR	60	80	61	6.0	34	54	1960–2004
Mendenhall et al. [17]	2004	42		57	42	56	43	6.6	NR	21	1966–2001
Silverman et al. [16]	2004	25		82	68	86	79	7.4	20	20	1971–2001
Balamucki et al. [13]	2012	73	Surgery + PORT	75	57	89	84	8.6	30 (T4)	20	1966–2008
Iseli et al. [14]	2009	93		76	57	73	44	9.0	40	54	1966–2007
Chen et al. [15]	2006	90		NR	65	92	84	5.0	52	31	1960–2004
Mendenhall et al. [17]	2004	56		77	55	94	91	6.6	NR	21	1966–2001
Silverman et al. [16]	2004	50		80	61	85	72	7.4	62	40	1971–2001
Present study	2021	24		85	68	83	83	5.0	29	58	1993–2017
Balamucki et al. [13]	2012	44	defRT (Photon)	56	37	55	36	8.6	73 (T4)	20	1966–2008
Iseli et al. [14]	2009	10		25	0	27	0	9.0	86	0	1966–2007
Present study	2021	20		80	66	83	33	7.6	75	10	1993–2017
Ikawa et al. [38]	2017	113	defRT (Carbon)	75	NR	69	NR	5.0	61 (T4)	NR	2006–2013
Shirai et al. [37]	2017	21		90 (3-yr)	NR	90 (3-yr)	NR	3.3	86	NR	2010–2014
Sulaiman et al. [36]	2018	289 *		94 (2-yr)	NR	88 (2-yr)	NR	2.5	85	12	2003–2014

* including 55 cases of postoperative macroscopic residual or postoperative recurrent tumor. PORT, post-operative radiotherapy; defRT, definitive radiotherapy; and MSG, major salivary gland; NR, not reported.

## 5. Conclusions

The standard treatment for adenoid cystic carcinoma of the head and neck is surgical resection followed by PORT. In our study, according to stratification of LC, PORT was favorable in T1/2/3 patients and defRT was favorable in T4 and stage IV patients. However, there were no significant differences in OS, DMFS, or PFS between PORT and defRT. Therefore, we consider that defRT for adenoid cystic carcinoma of the head and neck could be another treatment option if appropriate cases such as unresectable or refusal of resection are selected.

## Figures and Tables

**Table 1:** Characteristics of the patients.

Characteristics	Surgery + PORT (*n* = 24)	defRT (*n* = 20)	*p* Value
Gender			0.5467
Male	11	7	
Female	13	13	
Age (years)			0.5164
Median	57	63	
Range	38–73	16–76	
Performance status			>0.9999
0	19	16	
1	5	4	
Histology–solid component			0.0758
Presence	10	2	
Absence	13	14	
Unknown	1	4	
Primary site			0.0014
Major salivary gland	14	2	
Others	10	18	
Oral cavity	6	5	
Nasal or paranasal cavity	2	8	
Pharynx	1	5	
Lacrimal sac	1	0	
Tumor stage			0.0121
T1/2/3	4/13/5	1/4/6	
T4	2	9	
Nodal stage			0.2172
N0	22	15	
N1/2	2/0	2/3	
Stage			0.0014
I/II/III	4/13/5	1/2/5	
IV	2	12	
Concurrent chemotherapy			0.0007
Yes	6	16	
No	18	4	
Radiotherapy (Gy)			0.0048
Median	60	66	
Range	60–80	50–80	

PORT, postoperative radiotherapy; defRT, definitive radiotherapy.

**Table 2:** Risk factors for overall survival and local control rates for all patients.

Variation		All Cases (*n* = 44)				
		*n*	5-yr OS	*p* Value	5-yr LC	*p* Value
Solid component	Absence	28	81.5		80.5	
	Presence	12	80.5	0.9983	81.5	0.8399
Primary site	MSG	16	93.8		80.8	
	others	28	77.5	0.4015	84.5	0.8098
Tumor stage	T1/2/3	33	86.9		86.9	
	T4	11	70.1	0.1238	72.7	0.0121
Nodal stage	N0	38	88.5		86.3	
	N1/2	6	44.4	<0.0001	60.0	0.1557
Stage	I/II/III	29	96.0		88.9	
	IV	15	57.8	0.0022	72.7	0.0062
Chemotherapy	No	22	85.5		86.1	
	Yes	22	80.8	0.8239	80.2	0.0644

OS, overall survival; LC, local control; MSG, major salivary gland.

**Table 3:** Comparison of overall survival and local control rates by treatment modality in patients with clinical factors.

Variation		Overall Survival					Local Control				
		Surgery + PORT		defRT		*p* Value	Surgery + PORT		defRT		*p* Value
		3-yr	5-yr	3-yr	5-yr		3-yr	5-yr	3-yr	5-yr	
Solid component	Absence	100	90.9	80.0	72.7	0.2288	84.6	84.6	86.7	75.8	0.0684
	Presence	90.0	77.1	100	100	0.4861	77.1	77.1	100	100	0.3797
Primary site	MSG	92.9	92.9	100	100	0.7055	77.9	77.9	100	100	0.5490
	Others	100	77.8	83.3	77.8	0.5205	90.0	90.0	88.9	81.5	0.0958
Tumor stage	T1/2/3	95.2	89.6	90.9	81.8	0.2978	89.9	89.9	90.9	80.8	0.0397
	T4	100	50.0	77.8	77.8	0.5339	0.0	0.0	88.9	66.7	0.0004
Nodal stage	N0	100	89.1	87.5	87.5	0.7637	86.1	86.1	93.8	86.5	0.0536
	N1/2	50.0	NR	75.0	50.0	0.4504	0.0	0.0	75.0	NR	0.3508
Stage	I/II/III	100	100	100	94.1	0.6609	89.7	89.7	100	87.5	0.0768
	IV	66.7	33.3	75.0	65.6	0.3570	33.3	NR	83.3	83.3	0.0193
Chemotherapy	Yes	83.3	62.5	100	87.1	0.8533	62.5	62.5	93.7	85.9	0.7894
	No	100	93.3	50.0	50.0	0.0509	88.5	88.5	75.0	75.0	0.4767

OS, overall survival; LC, local control; PORT, post-operative radiotherapy; defRT, definitive radiotherapy; MSG, major salivary gland; NR, not reached.

**Table 4:** Acute and late adverse events for all patients.

Variation	Grade 2 (%)	Grade 3 (%)	Grade 4 (%)
Acute adverse event			
Mucositis	18 (41)	5 (11)	0 (0)
Dermatitis	11 (25)	0 (0)	0 (0)
Dysgeusia	3 (7)	0 (0)	0 (0)
Dry mouth	1 (2)	0 (0)	0 (0)
Dysphagia	1 (2)	0 (0)	0 (0)
Late adverse event			
Dry mouth	1 (2)	0 (0)	0 (0)
Trismus	1 (2)	0 (0)	0 (0)
Retinopathy	1 (2)	0 (0)	0 (0)
Oral hemorrhage	1 (2)	0 (0)	0 (0)
Keratitis	0 (0)	1 (2)	0 (0)

## Data Availability

The data presented in this study are available on request from the corresponding author.
